# Communicating Science: A Shared Responsibility

**DOI:** 10.21315/mjms2018.25.5.1

**Published:** 2018-10-30

**Authors:** Khayriyyah Mohd Hanafiah

**Affiliations:** School of Biological Sciences, Universiti Sains Malaysia, 11800 USM, Pulau Pinang, Malaysia

**Keywords:** STEM, communication, public engagement, sustainability

## Abstract

Malaysia faces increasing alienation of science in the community. While this is a global and multifaceted issue, science communication plays a pivotal role in making science more intuitive for the general public. Scientific communication requires brevity and accuracy—targeted to an interested audience hungry for details. Conversely, science communication requires conveying the bigger picture with clarity and impact—targeted to an audience that needs to be courted by an idea. The challenge for scientists and academics is to find a balance between details sufficient to carry the scientific “truth”, while appeasing the human desire for ease and simplicity. Critically, science communication is a powerful device to tackle the increasingly urgent challenge of sustaining scientific progress in a post-truth era. Here, I discuss the role of scientists, key elements of science communication, and propose instruction of Philosophy of Science and debate to equip scientists with the crucial skills required for impactful science communication.

## Introduction

For many researchers, the dreaded question at parties and family gatherings is perhaps “What is your research about?” Indeed, nothing kills a casual conversation faster than data overload and technical jargon, which builds a barrier in communication. Unfortunately, this has become ingrained in how many of us perceive scientific research. This may be one factor why the public can discuss politics, appreciate art and music, and even analyse gastronomy, but matters concerning STEM (Science, Technology, Engineering and Mathematics) remain confined to conversations among peer researchers. This lack of relatability and accessibility of science in the public sphere has broad ramifications. Since 1998 under 50% of Malaysians surveyed are able to answer basic scientific questions, while more than 50% believed that pseudoscience such as astrology were scientific (1). Harmful anti-vaccination campaigns in Malaysia have gained traction and led to 340% increase in vaccine-preventable diseases such as measles in 2016 (2, 3).

As science becomes further alienated from public interest, fewer investments are made, limiting potential for impactful innovations. Job opportunities in STEM become scarce (4), restricted only to technical positions in research and development, with fewer roles in media, education and policy development. Consequently, Malaysia has witnessed a reduction in students entering the science stream, totalling only 23.5% of upper secondary school students enrolled in 2017 (5). With this diminishing pool of students, Malaysia is predicted to face a crisis in supply of STEM practitioners in the future. While Malaysia aspires to nurture a knowledge-based society, public engagement is rarely prioritised for many scientists, and is left instead to select individuals, often without scientific training, to champion. It is ironic that this widespread dismissiveness in communicating science to a public audience, in order to chase more pressing scientific endeavours, may have led to diminishing resources and relevance, which now threatens sustainability of our scientific endeavours.

Considering that science communication plays a critical role in longevity of science, I discuss the role of scientists in the community, present elements of effective science communication as well as propose for inclusion of Philosophy of Science and debate as key aspects of scientific training, in order to arm future scientists with the propensity and ability to exchange ideas with everyone.

### What are the Role and Responsibilities of Scientists?

**Figure 1:**
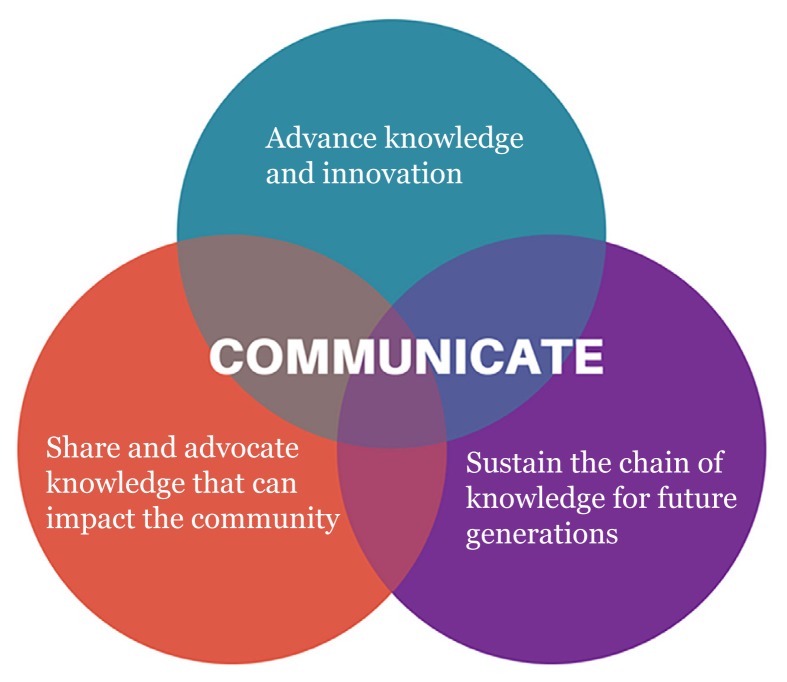
Role and responsibilities of scientists (interchangeably used with the more broad “researchers in STEM fields”)

Most scientists are aware of our immediate role to advance knowledge and innovation (Figure 1). This role often centres on planning and conducting experiments, analysing and interpreting data, and communicating with our peers. We also have explicit obligations to train future scientists, and this is often fulfilled through our complementary role as lecturers communicating with students in universities. However, we often neglect the more abstract responsibilities that we have as role models and advocates in our communities. Engaging with the public is seen as a distant priority, a positive thing but not really expected as part of our core duties.

In this post-truth era, with the exponential rise in information at everyone’s fingertips and anti-scientific narratives packaged in “fake news” or “conspiracy theories”, holding our roles as opinion-shapers at arm’s length may be to the detriment of scientific progress. One of the key drivers of successful control in deadly diseases such as HIV/AIDS was the joint advocacy by opinion leaders, patients and the research community, which led to higher visibility and increased global investment. A mere few decades since its discovery in 1981, the effort to control HIV resulted in many new drugs and diagnostic tools, which has transformed this disease from being fatal to a chronic condition (6). Alarmingly, progress in control of tuberculosis and measles, which have plagued humanity for centuries, may be decelerating—even reversed—owing to complacency, a lack of visibility, and rampant misconceptions e.g. controversies surrounding vaccination (7). In addressing issues of misinformation, scientists are in the best position to share up-to-date relevant knowledge, while simultaneously upholding evidence-based practices within the community. If we accept that science communication is part of our core responsibilities, this can be as casual as chatting with a neighbour, or more concerted such as through outreach and media engagement. More importantly, without artfully asserting our stand as scientists, we relinquish our authority in scientific discourse concerning politically intertwined issues such as evolution, vaccination, and climate change to influential spokespeople with potentially non-scientific views (8).

### What is the Difference between Scientific Communication and Science Communication?

**Figure 2:**
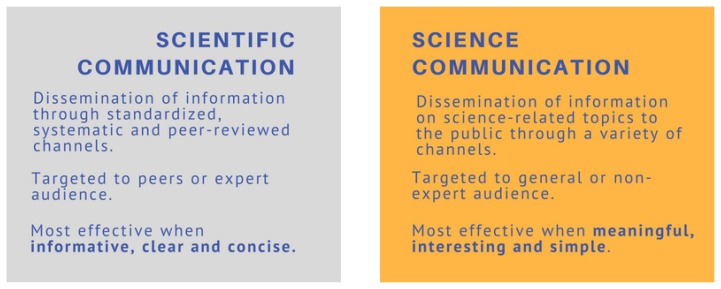
Comparison of scientific communication and science communication

Existing definitions of scientific and science communication appear broad and overlapping, but for the purpose of this discussion, they are distinguished as separate activities. The common goal relates to disseminating science-related information, with the critical difference being the target audience (Figure 2).

The majority of researchers engage in scientific communication through preparing manuscripts, grant proposals and conference presentations. For these, the target audience possess background knowledge and “speak the same language”, allowing us to focus on preparing detailed, clear and well-substantiated content. However, method of delivery is often held to lower standards, and an otherwise tedious lecture can appear fascinating to an initiated and receptive audience. The problem arises when scientists neglect the fact that the public is often an uninitiated and disinterested audience, and tries only to reach them with facts and figures. In Advice to Lecturers written nearly two centuries ago, Faraday’s (9) most striking point is perhaps the following:

“The most prominent requisite to a lecturer [....] is a good delivery; for though to all true philosophers, science and nature will have charms innumerable in every dress, yet I am sorry to say that the generality of mankind cannot accompany us one short hour unless the path is strewed with flowers.”

Today, with the ever-decreasing human attention span, the “short hour” of Faraday’s time may need to be revised to four minutes—the average duration of popular YouTube videos (10).

To “strew the path with flowers”, unnecessary details must be removed and key messages packaged in creative ways for anyone to relate and/or find relevance to their daily lives. Often scientific research takes us down narrow paths with increasingly specific questions. Talking to a non-expert audience forces us to ask bigger-picture questions about our science such as: What is the point? Why is it important? How is it interesting? Once the key messages are identified, organising flow of content, akin to the structure of an unfolding story—with a beginning, middle, and end—is crucial to sustain audience engagement. Analogies are also invaluable in helping the audience connect new ideas with pre-existing knowledge. Ideally, these analogies are not just parallel scenarios, but scenarios that evoke emotion, which then adds meaning to the content. Finally, to fulfill the human need for closure, the story must be satisfactorily concluded, either with a punchline, or returning to where it began.

### How do We Prepare Future Scientists? A Case for Philosophy of Science and Debate

Confusing science communication as “dumbing it down” is erroneous and such presumptions of superiority only sustain the gap between scientists and everyone else. The act of simplifying complex ideas and presenting them in engaging language that speaks to everyone requires a fundamental and philosophical understanding of the subject—a reorientation in perspective. Without initiation early in scientific training, this may prove challenging for even very experienced researchers. Instructions in Philosophy of Science and debate, which focus on scientific critique, logical argumentation and public speaking to complement rigorous training in scientific methods may better prepare future scientists for science communication.

Philosophy of Science, which contends with questions such as “What is science?” and exposes students to history and evolution of scientific methodology (11), enables them to grasp the implications of their observations, enhancing their ability to connect the dots and to envision the bigger picture, even when studying nature at the femtoscale. Without understanding the philosophy underlying human scientific advancement, science is doomed to be narrowly perceived as a collection of concrete facts and figures, instead of a human iterative quest for the truth.

Inevitably, many important scientific issues are inherently intertwined with political, cultural and religious issues. Topics in science that are controversial such as vaccines, animal testing, embryonic stem cell research, climate change, genetically modified organisms, evolution, and alternative medicine may be less polarising if more scientists are armed with basic skills in debate (8). To persuade an audience, debate harnesses the three components of rhetoric: ethos—speaker credibility, pathos—emotional appeal, and logos—line of logic (12). More importantly, debaters are trained in the art of listening to opposing views—to highlight the points of consensus, address points of contention and reassert their positions of strength. A better understanding of how different people perceive and respond to information, combined with a balance of logical argumentation, effective storytelling and credible persona, may not only address potentially harmful views that challenge scientific integrity and progress, but also reaffirm views that are aligned with scientific evidence.

## Conclusion

Science communication is imperative in our quest to sustain scientific progress and human capital in STEM, ultimately to materialise national aspirations of Malaysia becoming a high-income and knowledge-based society. Limiting our responsibilities as scientists to the practice of science and scientific communication, while avoiding communication with the public comes at the peril of an increasingly science-illiterate, misinformed society. With adequate exposure, encouragement and empowerment more STEM practitioners can adopt an active and effective role in science communication.
